# Assessment of Type 2 Diabetes Risk Among Nurses Working in the Emergency Department

**DOI:** 10.1002/nop2.70552

**Published:** 2026-04-15

**Authors:** Alpaslan Açikgöz, Sema Aytaç, Özlem Ovayolu

**Affiliations:** ^1^ Çukurova State Hospital, Adana Provincial Directorate of Health Adana Türkiye; ^2^ Department of Nursing Gaziantep University Gaziantep Türkiye

**Keywords:** diabetes risk, emergency department, FINDRISK, nurse

## Abstract

**Aim:**

This study aimed to assess the risk of type 2 diabetes mellitus (DM) among emergency department nurses.

**Design:**

The study has a cross‐sectional design.

**Methods:**

A screening model was used to assess the risk of type 2 DM among nurses working in the emergency departments of public institutions in a province. Data were collected using a descriptive information form and the Finnish Diabetes Risk Questionnaire (FINDRISK). FINDRISK is an eight‐item questionnaire used to assess DM risk, with a minimum score of 0 and a maximum score of 26. Risk categories are defined as follows: very high (≥ 20 points), high (15–20 points), moderate (12–14 points), mild (7–11 points) and low (< 7 points). Higher scores indicate greater risk.

**Results:**

The mean age of emergency department nurses was 33.8 ± 7.7 years. Among the participants, 59.4% were male, 23.8% had worked in the emergency department for > 10 years, 68.9% reported frequent consumption of fast food meals, and only 12.2% engaged in regular physical activity. Additionally, 53.8% had not undergone DM screening, 64.5% did not monitor their lipid profile, and 65% did not monitor their blood pressure. According to the FINDRISK assessment, 58.3%, 30% and 4.4% of nurses were in the low‐, mild‐ and high‐risk groups, respectively, with a mean FINDRISK score of 6.74 ± 4.09. Nurses who had worked in the emergency department for ≥ 10 years, did not engage in physical activity, had chronic diseases or a history of high blood glucose levels, or had undergone DM screening exhibited significantly higher DM risk scores (*p* < 0.05).

**Conclusion:**

Emergency department nurses face varying degrees of DM risk influenced by individual factors. Therefore, assessment of DM is recommended, along with support to promote healthy lifestyle habits.

## 1 Introduction

Diabetes mellitus (DM) is a chronic metabolic disease characterised by hyperglycemia (Turkish Society of Endocrinology and Metabolism 2023). According to the International Diabetes Federation (2021), 10.5% of the adult population aged 20–79 years has DM, with nearly half unaware of their condition. Over 90% of the population is diagnosed with type 2 DM owing to socioeconomic, environmental and genetic factors (International Diabetes Federation 2021), and risk factors include age, sex, unhealthy diet, insufficient physical activity, obesity, hypertension and dyslipidemia (Turkish Society of Endocrinology and Metabolism 2023, 2022). Individuals may be exposed to these risk factors through their lifestyle and working conditions. As continuous care providers, nurses are particularly exposed to long working hours, irregular lifestyles, unhealthy eating habits and occupational stress. These factors adversely affect nurses' overall health and contribute to the development of various health problems (Viklund et al. 2023). Emergency departments—where urgent medical treatment and care are provided—are particularly high‐risk units owing to high patient volume and time‐sensitive demands (Nart and Kanan 2024). Given the demanding working conditions of emergency departments and the associated unhealthy dietary habits, emergency department nurses are at an increased risk of developing type 2 DM. In a study examining the prevalence of pre‐DM and DM among hospital staff in Turkey, 78.4% of employees—including physicians, nurses/midwives, secretaries and cleaning personnel—had glycated haemoglobin (HbA1c) levels ≤ 5.7%, and 38.7% were classified as overweight based on body mass index (BMI). In the same study, 10.7% of those who were obese, 4.3% of those who were overweight, and 10.1% of those with hypertension had HbA1c levels ≥ 6.5% (Ak and Utlu 2023). Shift work has also been associated with an increased DM risk (Viklund et al. 2023). Previous studies have consistently indicated that night shift work is associated with negative health consequences, notably a high risk of type 2 DM (Xie et al. 2024; Hemmer et al. 2021). Emergency department nurses constitute a population at increased risk of metabolic disorders as a result of shift work, high occupational stress, and disrupted lifestyle patterns. Nevertheless, existing literature predominantly addressed nurses as a broader group, with a marked paucity of evidence specifically examining DM risk among emergency department nurses. This underscores a critical gap in existing knowledge. Accordingly, this study aimed to assess the risk of type 2 DM in this population.

## 2 Materials and Methods

### 2.1 Study Population and Sample

This descriptive, cross‐sectional study was conducted between July and October 2023 to assess the risk of type 2 DM among nurses working in the emergency departments of public institutions in a province. Nurses who had been working in the emergency department for at least 3 months, were ≥ 18 years old, and voluntarily agreed to participate were enrolled (Turkish Society of Endocrinology and Metabolism 2023; International Diabetes Federation 2021; Viklund et al. 2023). Nurses who did not meet the inclusion criteria, diagnosed with type 2 DM, or declined to participate (*n* = 8) were excluded.

All nurses working in the emergency department who met the inclusion criteria were invited to participate in the study, whereas those who did not meet the criteria or declined participation (*n* = 8) were excluded. The sample size was calculated using Cochran's formula (*n*_0_ = *Z* ^2^pq/*d* ^2^), with a 95% confidence level (*Z* = 1.96), a margin of error of 5% (*d* = 0.05) and an assumed proportion of *p* = 0.5 (Albelbeisi et al. 2024). Given that the target population was limited to 214 individuals, a finite population correction was applied, resulting in a minimum required sample size of 138 participants. To account for potential data loss, the sample size was increased by 10%–15%, yielding a target of 152–159 participants. During the study period, a total of 180 nurses were recruited, and subsequent analyses were conducted based on this sample.

### 2.2 Data Collection Tools

Data were collected using a descriptive information form assessing nurses' demographic and professional characteristics—including age, sex, educational background, length of service in the emergency department and duration of chronic illness—as well as the Finnish Diabetes Risk Score (FINDRISK).

#### 2.2.1 Descriptive Information Form

This form included items on nurses' age, sex, education level, years of employment in the emergency department, smoking and alcohol consumption, family history, dietary habits during shifts, history of DM, blood pressure and lipid monitoring and history of macrosomic childbirth (Viklund et al. 2023; Nart and Kanan 2024; Ak and Utlu 2023). The descriptive information form comprised items on sociodemographic, occupational and lifestyle‐related characteristics. Although formal psychometric validation is considered unnecessary for descriptive variables, the instrument was pilot‐tested on 20 individuals to ensure clarity and comprehensibility (Sukserm 2024).

#### 2.2.2 FINDRISK

FINDRISK is an eight‐item questionnaire used to assess DM risk by providing a 10‐year type 2 DM risk score, ranging from a minimum of ‘0’ to a maximum of ‘26’. Risk is categorised very high (> 20 points), high (15–20 points), moderate (12–14 points), mild (7–11 points) and low (< 7 points) (Lindström and Tuomilehto 2003). The questionnaire, recommended by the International Diabetes Federation for community‐based DM screening, was translated into Turkish by the Turkish Society of Endocrinology and Metabolism and is endorsed for use in adult DM screening (Turkish Society of Endocrinology and Metabolism 2022).

### 2.3 Data Collection Procedure

Data collection forms were administered through face‐to‐face interviews by the researcher in the respective emergency departments, with each interview lasting approximately 10 min.

### 2.4 Ethical Considerations

This study was conducted in accordance with the ethical principles of the Declaration of Helsinki and received approval from the relevant ethics committee (185/2023) and the institution where the study was conducted. Informed consent was obtained from all participating nurses. This study has been reported following the Strengthening the Reporting of Observational Studies in Epidemiology (STROBE) checklist for cross‐sectional studies.

### 2.5 Data Analysis

FINDRISK results were evaluated as frequencies and percentages according to risk categories. Comparisons between the variables included in the descriptive information form and FINDRISK scores were performed using independent samples *t*‐test, one‐way analysis of variance (ANOVA), Mann–Whitney *U* test and Kruskal–Wallis test, as appropriate. Spearman's correlation analysis was used to assess the associations between variables. Factors predicting FINDRISK scores were examined using simple linear regression analysis. Data were analysed using SPSS Statistics version 25.0 (IBM Corp., Armonk, NY, USA), and a *p*‐value of < 0.05 was considered significant.

## 3 Results

### 3.1 Sociodemographic Characteristics and FINDRISK Scores

The mean age of the emergency department nurses was 33.8 ± 7.7 years, 59.4% were male, 40.5% were female, 57.7% were married and 75.0% lived with family members. Additionally, 48.3% reported that their income matched their expenses, 54.4% were smokers and 28.3% consumed alcohol. By FINDRISK scores, the mean scores were 7.1 ± 5.0 (low risk) among male nurses, 7.8 ± 4.3 among married participants, 7.3 ± 4.5 (low risk) among those living with family members, and 7.9 ± 5.0 (low risk) among those whose income was less than their expenses (Table 1).

**TABLE 1:** Comparison of sociodemographic characteristics and FINDRISK scores of emergency department nurses.

Characteristics	*n* (%)	FINDRISK score (mean ± SD)	*p*
Age	33.8 ± 7.7 years		
*Sex*			
Female	73 (40.5)	6.2 ± 3.6	0.13
Male	107 (59.5)	7.1 ± 5.0	
*Marital status*			
Married	104 (57.7)	7.8 ± 4.3	**0.001**
Single	76 (42.3)	5.3 ± 4.4	
*Cohabitants*			
Living alone	45 (25)	5.2 ± 4.1	**0.001**
Living with family members	135 (75)	7.3 ± 4.5	
*Income status*			
Income more than expenses	34 (18.8)	5.6 ± 4.1	**0.03**
Income equivalent to expenses	87 (48.3)	6.4 ± 4.1	
Income less than expenses	59 (32.9)	7.9 ± 5.0	
*Education status*			
High school	10 (5.6)	9.30 ± 4.47	0.52
Associate degree	19 (10.6)	8.68 ± 6.73	
Undergraduate degree	140 (77.8)	6.19 ± 4.09	
Postgraduate degree	11 (6.0)	8.09 ± 3.04	
*Smoking*			
Yes	98 (54.4)	7.17 ± 4.78	0.53
No	79 (43.9)	6.13 ± 4.40	
Ex‐smoker	3 (1.7)	8.66 ± 0.57	
*Alcohol use*			
Yes	51 (28.3)	6.88 ± 1.32	0.90
No	128 (71.1)	6.66 ± 1.58	
Ex‐drinker	1 (0.6)	3.00 ±	
Total	180 (100)	6.74 ± 4.09	

*Note:* Bold values denote statistical significance at *p* < 0.05.

### 3.2 Comparison of FINDRISK Scores Based on Sociodemographic Characteristics

Among the participants, 38.3% had worked as nurses for 0–5 years, 45.5% consumed fruit occasionally, 60.0% consumed vegetables occasionally, and 68.9% reported frequent consumption of fast food meals while on duty. The mean BMI was 24.4 ± 3.6 kg/m^2^. The mean daily sleep duration was 6.9 ± 1.6 h for those working in shifts compared with 7.28 ± 2.05 h for those with regular working hours. Only 12.2% of the nurses engaged in regular physical activity, whereas 53.8% had not undergone DM screening, 64.5% had not had a lipid profile check, and 65.0% had not had their blood pressure measured.

Regarding FINDRISK scores, the mean scores were 9.11 ± 4.07 (low risk) among those with ≥ 10 years of emergency department experience, 7.19 ± 4.43 (low risk) among those not engaging in regular physical activity, 11.11 ± 5.45 (low risk) among those with chronic diseases, 7.81 ± 4.47 (low risk) among those who had undergone DM screening, and 15.42 ± 4.92 (high risk) among those with a history of high blood glucose levels. Furthermore, nurses with ≥ 10 years of emergency department experience who often consumed fast food meals while on duty, did not engage in regular physical activity, had a history of high blood glucose levels and blood pressure, had undergone lipid and blood pressure screening, had another chronic disease, and had a family history of DM exhibited significantly higher mean DM risk scores (Table 2) (*p* < 0.05).

**TABLE 2:** Analysis of diabetes risk scores based on specific characteristics of emergency department nurses.

Characteristics	*n* (%)	FINDRISK score (Mean ± SD)	*p*
*Work experience (years)*			
0–5	69 (38.3)	4.59 ± 3.60	**0.001**
6–10	38 (21.1)	6.31 ± 3.58	
11–15	18 (10)	5.44 ± 3.85	
16+	55 (30.6)	10.16 ± 4.49	
*Experience in the emergency department (years)*			
0–3	58 (32.3)	4.81 ± 3.61	**0.001**
4–7	73 (40.6)	6.76 ± 4.27	
7–10	6 (3.3)	8.16 ± 8.81	
10+	43 (23.8)	9.11 ± 4.07	
*Shift work status*			
Yes	173 (99.7)	6.72 ± 4.57	**0.001**
No	7 (0.3)	7.28 ± 2.05	
*Chronic disease*			
Yes	17 (9.5)	11.11 ± 5.45	**0.001**
No	163 (90.5)	6.28 ± 4.14	
*Dietary habits in the emergency department*			
Healthy	56 (31.1)	6.51 ± 4.36	0.65
Frequent consumption of fast food meals	124 (68.9)	6.84 ± 4.57	
*Social dietary habits*			
Healthy	112 (62.2)	6.76 ± 4.53	**0.001**
Frequent consumption of fast food meals	68 (37.8)	6.70 ± 4.47	
*Regular exercise and activity*			
Yes	22 (12.2)	3.50 ± 3.58	**0.001**
No	158 (87.7)	7.19 ± 4.43	
*Previous health checkup for diabetes*			
Yes	83 (46.2)	7.81 ± 4.47	**0.001**
No	97 (53.8)	5.82 ± 4.33	
*Previous history of high blood glucose levels (n = 83)*			
Yes	7 (3.9)	15.42 ± 4.92	**0.001**
No	76 (42.2)	7.11 ± 3.74	
No information	97 (53.8)	7.81 ± 4.47	
*Previous lipid monitoring*			
Yes	64 (35.5)	7.93 ± 4.72	**0.001**
No	116 (64.5)	6.08 ± 4.24	
*Previous blood pressure monitoring*			
Yes	63 (35)	8.22 ± 4.94	**0.001**
No	117 (65)	5.94 ± 4.04	
*History of high blood pressure (n: 63)*			
Yes	13 (7.2)	12.46 ± 5.44	**0.001**
No	50 (27.7)	7.15 ± 4.16	
No Information	117 (65.1)	4.45 ± 4.36	
*History of macrosomic childbirth*			
Yes	4 (2.2)	7.50 ± 2.38	**0.001**
No	55 (30.6)	8.47 ± 4.90	
I don't have children	121 (67.2)	5.93 ± 5.93	
*Family history of diabetes*			
Yes	77 (42.8)	9.25 ± 3.96	**0.001**
No	103 (57.2)	4.86 ± 3.93	
Total	180 (100)	6.74 ± 4.09	

*Note:* Bold values denote statistical significance at *p* < 0.05.

### 3.3 Correlation Between FINDRISK Scores and Other Parameters

A positive correlation was observed between the FINDRISK score and age, weight, BMI, shift work duration, years of emergency department experience, total years in nursing and daily coffee consumption (Table 3) (*p* < 0.05).

**TABLE 3:** Correlation between FINDRISK scores and other parameters.

	FINDRISK total score	
	Correlation coefficient (*r*)	*p*
Age	0.57	**0.001**
Weight	0.33	**0.001**
BMI	0.52	**0.001**
Shift work duration (years)	0.47	**0.001**
Work experience in the emergency department (years)	0.34	**0.001**
Overall work experience (years)	0.53	**0.001**
Frequency of physical activity	−0.46	0.38
Activity duration	−0.15	0.52
Daily fruit consumption	0.19	0.18
Daily coffee consumption	0.79	**0.001**

*Note:* Bold values denote statistical significance at *p* < 0.05.

### 3.4 Distribution of Emergency Department Nurses by DM Risk Category

According to FINDRISK scores, 58.3%, 30.0% and 4.4% of nurses were in the low‐, mild‐ and high‐risk groups, respectively, with a mean score of 6.74 ± 4.09 (very low risk) (Table 4).

**TABLE 4:** Distribution of emergency department nurses across diabetes risk categories.

	*n* (%)	FINDRISK score (mean ± SD)
Low	105 (58.33)	3.78 ± 4.62
Mild	54 (30)	9.12 ± 3.78
Moderate	11 (6.11)	13.09 ± 3.14
High	8 (4.44)	16.75 ± 1.71
Very high	2 (1.12)	23 ± 0.00
Total	180 (100)	6.74 ± 4.09

### 3.5 Simple Linear Regression of Sociodemographic, Occupational and Lifestyle Predictors

Simple linear regression analyses revealed significant positive associations between the FINDRISK score and age (*B* = 0.96, *p* < 0.001), BMI (*B* = 0.40, *p* < 0.001), shift work duration (*B* = 1.02, *p* < 0.001) and work duration in the emergency department (*B* = 0.27, *p* < 0.001) (Table 5).

**TABLE 5:** Simple Linear Regression Analyses of Sociodemographic, Occupational and lifestyle factors predicting the FINDRISK score.

Variables	*B*	Std. Error	*β*	*t*	*p*	*R* ^2^	Durbin–Watson
Age	0.96	0.11	0.565	9.13	< 0.001*	0.315	1.54
Body weight	0.94	0.20	0.333	4.70	< 0.001*	0.111	2.00
Body mass index	0.40	0.05	0.523	8.19	< 0.001*	0.523	1.95
Duration of shift work	1.02	0.22	0.473	4.59	< 0.001*	0.224	1.83
Duration of work in the emergency department	0.41	0.07	0.340	5.79	< 0.001*	0.116	1.91
Professional working duration	0.74	0.17	0.527	4.38	< 0.001*	0.278	1.51
Physical activity duration	−0.29	0.20	−0.110	−1.47	0.140	0.012	1.83
Daily fruit consumption	0.02	0.01	0.140	2.01	0.045*	0.020	1.75
Daily coffee consumption	0.01	0.01	0.120	2.63	0.009*	0.100	2.13
Previous diabetes screening	−0.02	0.01	−0.220	−3.03	0.003*	0.220	2.00
Average daily sleep duration	−0.01	0.02	−0.030	−0.41	0.680	0.000	1.97
Type of nutrition in the emergency department	0.01	0.01	0.030	0.45	0.650	0.030	1.81
Type of nutrition in social life	−0.01	0.01	−0.010	−0.08	0.940	0.000	1.75

*Note:* Dependent variable: FINDRISK score. Each predictor was entered separately into the model (simple linear regression). *Statistically significant at *p* < 0.05.

Abbreviations: B = unstandardised regression coefficient; *β* = standardised regression coefficient.

## 4 Discussion

Emergency departments operate 24 h a day, 7 days a week, using shift or on‐call systems to ensure uninterrupted service (Nart and Kanan 2024). Working in shifts or on‐call rotations disrupts the body's natural biological rhythms, potentially leading to a range of physiological and psychological disturbances (Viklund et al. 2023). Shift work affects individuals physically, mentally, and socially and demanding work schedules contribute to the development of various health problems (Sooriyaarachchi et al. 2022). In particular, night‐time sleep deprivation among shift workers reduces alertness and impairs cognitive performance. These individuals are also reported to have higher rates of gastrointestinal and cardiovascular diseases, weight fluctuations, unhealthy dietary habits, physical inactivity and excessive consumption of caffeine, alcohol and tobacco (Sooriyaarachchi et al. 2022). However, very few studies have specifically examined DM risk among emergency department nurses. In the study by Sánchez‐Jiménez, 59% of nurses were found to have a high or very high risk of type 2 DM, and a very high risk score was associated with high HbA1c, glucose and insulin levels and insulin resistance (Sánchez‐Jiménez et al. 2019). In another study, 42.7% of nurses were at low risk, 38.4% at mild risk, 10.8% at moderate risk, 7.6% at high risk, and 0.5% at very high risk of DM (Abdullah et al. 2019). In the present study, 58.3% of emergency department nurses were at low risk, 30.0% at mild risk, and 4.4% at high risk, and the mean FINDRISK score indicated a very low DM risk. These findings demonstrate that emergency department nurses experience DM risk at varying levels and that those exhibiting high risk profiles warrant closer clinical surveillance. The risk factors identified in the present study are well‐established contributors to DM development, thereby reinforcing the clinical significance of the observed outcomes. Consequently, systematic assessment of DM risk among emergency department nurses is imperative to safeguard their physical, psychological, and social well‐being and ensure the sustained quality of healthcare delivery.

When combined with the occupational hazards inherent in nursing, nurses' work‐related and social habits may contribute to the development of chronic diseases. In particular, shift work has been associated with high obesity risk (Özpak Akkuş and Mermer 2022). Although irregular diets, sedentary lifestyles and stress are well‐established contributors to DM, additional risk factors include high BMI, blood pressure and glucose levels; physical inactivity; insufficient intake of fruits, vegetables and whole grains; a family history of DM; shift work; existing chronic diseases; and lack of regular health screenings (Tárraga Marcos et al. 2025; Melnyk et al. 2020; Birgül et al. 2024; Ezber et al. 2022). Abdullah et al. (2019) identified correlations between DM risk and high blood glucose levels, family history, dietary patterns and antihypertensive medication use and advocated for frequent monitoring of high‐risk individuals (Abdullah et al. 2019). Another study reported that despite health workers' awareness of the benefits of exercise, a substantial proportion did not adhere to recommended physical activity guidelines, and insulin resistance was more prevalent among them (Melnyk et al. 2020). According to Di Lorenzo et al. (2021) adherence to a Mediterranean diet may help mitigate the negative effects of occupational stress and irregular eating patterns. They emphasised that most healthcare workers, particularly those working long or night shifts, tend to adopt less healthy diets, which may exacerbate insulin resistance (Di Lorenzo et al. 2021). A systematic meta‐analysis revealed that adherence to the Mediterranean diet is inversely associated with insulin resistance, even among individuals with a genetic predisposition to type 2 DM (Papadaki et al. 2020). In Vural Doğru's study examining DM risk among relatives of patients with type 2 DM, increases in BMI and waist circumference were associated with higher FINDRISK scores and high risk levels. Additionally, relatives of patients who did not engage in regular physical activity were more likely to belong to the high‐risk group for type 2 DM (Birgül et al. 2024). In the present study, higher mean DM risk scores were observed among nurses who had longer professional experience and extended tenure in the emergency department, those who had comorbid chronic conditions, those who did not engage in regular physical activity, those who had previously identified elevations in blood glucose, blood pressure, or lipid levels, nurses who frequently consumed fast food meals while on duty, and those who had a family history of DM. A significant positive correlation was found between DM risk scores and age, BMI, daily coffee consumption, shift work, work duration in the emergency department, and overall professional experience. The association of higher FINDRISK scores with longer working duration, presence of chronic illness, physical inactivity, unhealthy dietary patterns and family history of DM indicates that DM risk is influenced by both individual characteristics and occupational factors. Furthermore, age, BMI and employment duration were identified as significant predictors of DM risk. Notably, age and BMI exerted strong effects (*β* > 0.5), whereas prolonged employment in the emergency department demonstrated a moderate positive effect (*β* = 0.34, *p* < 0.001), consistent with risk exposures such as irregular eating habits, night shifts and occupational stress. Additionally, participants who had previously undergone DM screening exhibited lower risk levels, supporting the protective role of early detection. Overall, our findings highlight age, BMI, employment duration and screening history as key determinants in DM risk assessment.

In conclusion, this study demonstrates that both individual and occupational factors play a critical role in determining DM risk. In particular, the development of targeted preventive strategies is essential for nurses with prolonged employment in emergency departments. The findings provide novel insights into the interaction between working conditions and metabolic health and offer practical implications for workplace health policies and early intervention programs. Moreover, implementing strategies that promote healthy lifestyle behaviors among emergency department nurses may substantially contribute to addressing modifiable risk factors for DM.

## 5 Conclusion and Recommendations

This study demonstrates that DM risk among emergency department nurses is influenced by both individual and occupational factors, including age, BMI, shift work duration and work duration in the emergency department. Notably, the increased risk observed among nurses with prolonged employment in the emergency department highlights the significant effect of working conditions on metabolic health. Furthermore, lower risk levels among participants who had previously undergone DM screening underscore the protective role of early detection and regular monitoring. In this context, regular assessment of health behaviours related to DM among emergendy department nurses is recommended, as well as providing support in assuming primary responsibility for the protection and promotion of their own health.

### 5.1 Limitations of the Study

This study has several limitations. First, the use of self‐reported data may have introduced recall and social desirability bias, particularly for variables such as physical activity, dietary habits and family history of DM. Second, the sample consisted solely of emergency department nurses from a single region in Turkey, which limits the generalisability of the findings to nurses working in other regions or different clinical units. Third, the assessment of type 2 DM risk was based solely on a single scale and was not corroborated with clinical or biochemical measures. Finally, the cross‐sectional research design precludes the establishment of causal relationships between risk factors and DM risk. To obtain stronger evidence, future studies are recommended to involve larger and more diverse samples, utilise multivariate analyses and employ longitudinal research designs.

## Author Contributions

Study design: A.A., S.A. and Ö.O. Data collection: A.A. Literature search: A.A., S.A. and Ö.O. Drafting manuscript: S.A., Ö.O. and A.A.

## Funding

This study received no specific grant from any funding agency in the public, commercial, or not‐for‐profit sectors. The article processing charge (APC) was supported by The Scientific and Technological Research Council of Türkiye (TÜBİTAK) under its Open Access Publication Support Program.

## Ethics Statement

This research was conducted in accordance with the ethical principles of the Declaration of Helsinki. Ethical approval was obtained from the institutional ethics committee (185/2023), as well as from the institution where the study was conducted and the participating nurses prior to the commencement of the study.

## Conflicts of Interest

The authors declare no conflicts of interest.

## Data Availability

The datasets used and/or analysed during the current study are available from the corresponding author on reasonable request.
